# The modified mRNA vaccine protects immunocompromised AG129 mice from lethal challenge and multi-tissue infection by Zika virus

**DOI:** 10.1080/22221751.2025.2556729

**Published:** 2025-09-03

**Authors:** Yuhuan Yan, Junbin Wang, Hao Yang, Yun Yang, Longhai Yuan, Cong Tang, Yanan Zhou, Qing Huang, Wenhai Yu, Xiaoming Liang, Dongdong Lin, Yanwen Li, Xuena Du, Yuxia Yuan, Rui Peng, Jiali Xu, Zhaolan Guo, Wenhao Xie, Wenqi Quan, Hongyu Chen, Jian Zhou, Shuaiyao Lu, Xiaozhong Peng

**Affiliations:** a Yunnan Key Laboratory of Cross-Border Infectious Disease Control and Prevention and Novel Drug Development, Institute of Medical Biology, Chinese Academy of Medical Sciences and Peking Union Medical College, Kunming, People’s Republic of China; b Institute of Basic Medical Sciences Chinese Academy of Medical Sciences, School of Basic Medicine Peking Union Medical College, Beijing, People’s Republic of China; c State Key Laboratory of Respiratory Health and Multimorbidity, Beijing, People’s Republic of China; d Key Laboratory of Pathogen Infection Prevention and Control (Peking Union Medical College), Ministry of Education, Beijing, People’s Republic of China; e Yunnan Provincial Key Laboratory of Vector-Borne Diseases Control and Research, Kunming, People’s Republic of China

**Keywords:** Zika virus, mRNA vaccine, lethal challenge, AG129 mice, antibody-dependent enhancement

## Abstract

The multiple epidemics of Zika virus (ZIKV) posed a substantial threat to public health. Clinical evidence suggests that ZIKV could break through the blood–brain, blood-placenta, and blood-testis barriers, leading to severe outcomes such as congenital malformations in newborns and Guillain–Barré syndrome in adults. Currently, there are no specific treatments for ZIKV infection. To address the antibody-dependent enhancement (ADE) of dengue virus (DENV) infection induced by ZIKV vaccination, we designed two modified prM-E RNAs (ZA and ZB) with specific mutations either shielding or disrupting the conserved fusion-loop epitope in the E protein. Then, we chose the mRNA-LNP vaccine platform to evaluate the safety and efficacy. After prime-boost immunization, ZA vaccine could induce high levels of T cells secreting IFN-γ and exhibit limited neutralizing ability against Asian-lineage and African-lineage ZIKV. After ZIKV challenge, ZA vaccine could provide complete protection in immunocompromised AG129 mice at low levels of neutralizing antibodies, preventing viral dissemination to the brain, uterus, and testes. Importantly, the ZA vaccine also reduced the ADE effect of DENV infection. Although ZB vaccine exhibited good immunogenicity, it could not achieve complete viral clearance in AG29 mice. Our findings suggested that the ZA vaccine could prevent both lethal ZIKV infection and DENV ADE induced by infection or vaccination.

## Introduction

The 2016 Zika virus (ZIKV) epidemic in Brazil garnered global attention due to its large-scale transmission [1]. Initially discovered in 1947 in Uganda’s Sentinel Rhesus monkeys, ZIKV infection was considered a mild and sporadic disease for decades [2,3]. Phylogenetic analyses indicate that ZIKV has evolved into two lineages, Asian and African [4]. Notably, ZIKV has appeared the evolutionary enhancement of its infectivity in Aedes aegypti mosquitoes [5]. In addition, the effects of ZIKV on humans during its transmission suggest that the K101R substitution in the C protein or the S139N substitution in the prM protein may increase its infectivity and pathogenicity [6,7]. Clinical evidence in recent years demonstrated ZIKV infection was associated with Guillain–Barré syndrome in adults and congenital malformations in infants [8–10]. Although global ZIKV cases have declined since 2017, low-level transmission persists in several countries in the Americas and other endemic regions [11]. Therefore, we should attach importance to the research on ZIKV.

ZIKV is a mosquito-borne orthoflavivirus with a genome organization and protein structure similar to dengue virus (DENV) [12]. Its single-stranded positive RNA genome encodes three structural proteins (C, prM, and E) and seven nonstructural proteins (NS1, NS2A, NS2B, NS3, NS4A, NS4B and NS5) [13]. The E protein mediates viral attachment and entry by interacting with host cell receptors [14]. Upon infection, viral RNA is released into the cytoplasm, initiating the biosynthesis of viral polyproteins, which are subsequently cleaved into structural and nonstructural proteins. The NS5 protein acts as RNA-dependent RNA polymerase (RdRp) to generate offspring RNA [15]. In the cytoplasm, the C protein first assembles with offspring RNA into the viral RNA-protein complex. Then, the complex enters the endoplasmic reticulum lumen and obtains the lipid membrane with prM and E protein to form immature ZIKV (immZIKV) [16]. The surface of immZIKV is covered with 180 copies of prM-E heterodimers. The prM-E dimers undergo a low-pH mediated rearrangement at the trans-Golgi network, where the prM protein is cleaved, resulting in the formation of mature infectious ZIKV [16,17]. The surface of the mature virion is covered with 180 copies of M-E heterodimers. It is noteworthy that E protein is the main target antigen of neutralizing antibodies [18].

Currently, there are no specific treatments for ZIKV infection, making vaccination the most effective strategy for prevention and control. Previous studies have defined a single ZIKV serotype and indicated that infection or vaccination with a single ZIKV strain can provide immune protection against multiple strains [19]. According to previous studies of immunity to ZIKV, prM and E proteins are often selected as target antigens of ZIKV vaccines [20–23]. However, an important safety concern for ZIKV vaccine is the antibody-dependent enhancement (ADE) effect between ZIKV and its antigenically related dengue virus (DENV). The conserved fusion loop epitopes (FLEs) in their E protein could induce poorly neutralizing cross-reactive antibodies, which are considered the source of ADE. To address this, we designed two antigen sequences, ZA and ZB, introducing amino acid mutations to stabilize E protein dimers or disrupt the FLE. The latter has already been proven effective in reducing the ADE effect in the mRNA vaccine platform [24]. In the subunit vaccine platform, the former could abrogate antibody-dependent enhancement of dengue infection [25]. Given the timely and effective response of mRNA vaccines to epidemic outbreaks, we chose the mRNA-lipid nanoparticle (LNP) platform to evaluate the safety and efficacy of the ZIKV vaccine.

## Materials and methods

### Animals, ethics and biosafety statement

All female C57BL/6 mice (aged 4∼6 weeks) were obtained from the Institute of Medical Biology, Chinese Academy of Medical Sciences (Kunming, China; Manufacturing licence: SYXK (DIAN) K2022-0006). Male and female AG129 mice (aged 4∼6 weeks) were obtained from the National Institutes for Food and Drug Control (NIFDC; Manufacturing licence: SCXK (JING) 2022-0002). All animal experiments were approved by the Institutional Animal Care and Use Committee of the Institute of Medical Biology, Chinese Academy of Medical Science (Ethics number: DWSP202108008). In this study, AG129 mice are the engineered C57BL/6 mice with the double-knockout of α/β and γ interferon receptors.

### Generation of modified mRNA and lipid nanoparticles (LNPs)

The mRNA was synthesized in vitro using the HiScribe® T7 mRNA Kit (NEB, E2080S), with UTP replaced by N1-Methyl-Pseudo-UTP (Novoprotein). The modified mRNAs incorporate a poly-A tail and 5′ and 3′ untranslated regions (UTRs) derived from the Pfizer-BioNTech coronavirus vaccine (BNT162b2). Additionally, the mRNAs encoded the signal peptide from human IgE (MDWTWILFLVAAATRVHS) and the prM and E proteins. We aligned over 300 amino acid sequences of ZIKV strains from Asian and African lineages for the conserved region. To reduce or eliminate the production of antibodies that enhance dengue infection, three mutations were introduced in the E protein of ZA (L107C, A264C, A319C) and four mutations in that of ZB (T76R, Q77E, W101R, L107C). We chose the conserved sequence without mutations as a control.

LNP was prepared following the Moderna coronavirus vaccine protocol. Lipids were dissolved in ethanol at a concentration of 12 mM at a molar ratio of 50:10:38.5:1.5 (SM102: DSPC: cholesterol: DMG-PEG-2000). Then, the lipid mixture was mixed with a 50 mM citrate buffer (pH 4.0) containing mRNA at an N/P ratio of 8:1 (lipids: RNA) using a microfluidic mixer (FluidicLab). The LNP formulations were then ultrafiltered and concentrated in 20 mM Tris-HCl buffer. All formulations were characterized for particle diameter (90–110 nm) and encapsulation efficiency (≥95%).

### Transfection and protein expression analysis

To detect the protein expression, Vero cells were transfected with the mRNA using MessengerMAX reagent (Thermo, LMRNA003). At 12 and 24 h post-transfection, cells were lysed with RIPA buffer for Western Blot analysis. The PVDF membrane was blotted with ZIKV polyclonal prM antibody (GeneTex, GTX133305), ZIKV polyclonal E antibody (GeneTex, GTX133314) and orthoflavivirus monoclonal 4G2 antibody (GeneTex, GTX57154) at a dilution ratio of 1:1000. Meanwhile, we chose HRP-conjugated secondary antibodies (Affinity bioscience) for Western Blot.

### Immunization and ZIKV challenge

We adopted a prime-boost vaccination strategy in C57BL/6 and AG129 mice. There were five female C57BL/6 mice (n = 5) and six male/female AG129 mice (n = 6) in each group. Mice were immunized intramuscularly with 2.5 and 5.0 μg of mRNA-LNPs. Booster immunizations were administered 21 days after the prime vaccination. Twenty-eight days post-boost, all AG129 mice were challenged subcutaneously with 10^3^ TCID_50_ of ZIKV strain GZ01 (GenBank: KU820898). Mice were sacrificed eight days post-challenge.

### Viral load

Viral RNA was quantified by RT-qPCR using TaqMan Fast Viral 1-Step Master Mix (Thermo Fisher, 4444432) on a CFX384 Touch Real-Time PCR Detection System (Bio-Rad, USA). Primers and probe targeting the ZIKV prM-E gene were used: Forward primer (5′-TTGGTCATGATACTGCTGATTGC-3′), reverse primer (5′-CCTTCCACAAAGTCCCTATTGC-3′) and probe (5′-FAM-CGGCATACAGCATCAGGTGCATAGGAG-BHQ1-3′). Primers and probe targeting the DENV-1 gene were used: Forward primer (5′-CGAAGCCAAAGAGGGACTAAA-3′), reverse primer (5′-TACAAGGTTCCTCTCCACAAAC-3′) and probe (5′-FAM-TTTAGCGGTTCCTCTCGACACTGC-BHQ1-3′). The reaction system consisted of 2.5 μL qPCR mix, 0.5 μL probe, 0.5 μL forward primer, 0.5 μL reverse primer, 2.5 μL RNA sample, and 3.5 μL water. The reaction conditions were: 50°C for 5 min, 95°C for 20 s, and 40 cycles of 95°C for 3 s and 60°C for 30 s. Viral copy numbers were calculated based on standard curves.

### In vitro ADE assay

Serum from C57BL/6 mice was inactivated at 56°C for 30 min. Serum from the same vaccine group was pooled and diluted with DMEM at a ratio of 1:32. DENV or ZIKV, at a multiplicity of infection (MOI) of 0.1, was incubated with diluted serum or 4G2 antibody at 37°C for one hour. The serum-virus mixture was then added to 5×10⁴ K562 cells and incubated at 37℃ for three days. After cells were centrifuged and washed twice with PBS, we detected viral infectivity by One Step TB Green RT-PCR kit (Takara, RR096A). Primers targeting the DENV-1 gene were used: Forward primer (5′-CAAAAGGAAGTCGTGCAATA-3′), reverse primer (5′-CTGAGTGAATTCTCTCTACTGAACC-3′). Primers targeting the DENV-2 gene were used: Forward primer (5′-ACAAGTCGAACAACCTGGTCCAT-3′), reverse primer (5′-GCCGCACCATTGGTCTTCTC-3′). The reaction system consisted of 6.25 μL PCR buffer, 0.75 μL HS Mix, 0.25 μL RTase Mix, 0.5 μL forward primer, 0.5 μL reverse primer, 2.25 μL water, and 2 μL RNA sample. The reaction conditions were: 42°C for 5 min, 95°C for 10 s, and 40 cycles of 95°C for 5 s, 55°C for 30 s and 72°C for 30 s. Viral relative infectivity was calculated based on the following method: normalization RFU = RFU_DENV_/RFU_Actin_; Relative infectivity = norRFUvaccine/norRFU_PBS_.

### In vivo ADE assay

Serum from C57BL/6 mice was inactivated at 56°C for 30 min. Groups (n = 3) of AG129 mice were administered 10 μg mAb 4G2 or 20 μl immune serum (in 200 μL 1× PBS) intraperitoneally, and challenged subcutaneously 2 h later, with DENV-1 (10^5^ TCID_50_/mouse).

### Passive immunization

We collected immune serum from C57BL/6 mice 21 days after booster vaccination for passive transfer studies. Pooled mouse immune serum was transferred into groups of 3 AG129 mice at 200 μL per mouse using the intraperitoneal route of administration without dilution. The serum from C57BL/6 mice of the PBS group was used as a control. Within 1 h post-transfer of immune serum, mice were challenged with 10^3^ TCID_50_ of ZIKV strain GZ01 (in 100 μL 1× PBS) subcutaneously. All mice were euthanized when the control group died of viral infection.

### Data processing and statistics

Statistical analysis was performed using GraphPad Prism (8.0.2). The following statistical tests were used in this study: Dunnett's multiple comparisons test and two-way ANOVA. Quantitative data were presented as mean ± SD. *P* values < 0.05 were considered significant, with significance levels denoted as follows: NS, not significant (*P* > 0.05); *P < 0.05; **P < 0.01; ***P < 0.001; and ****P < 0.0001.

## Results

### mRNA design and in vitro protein expression

**Figure 1.**
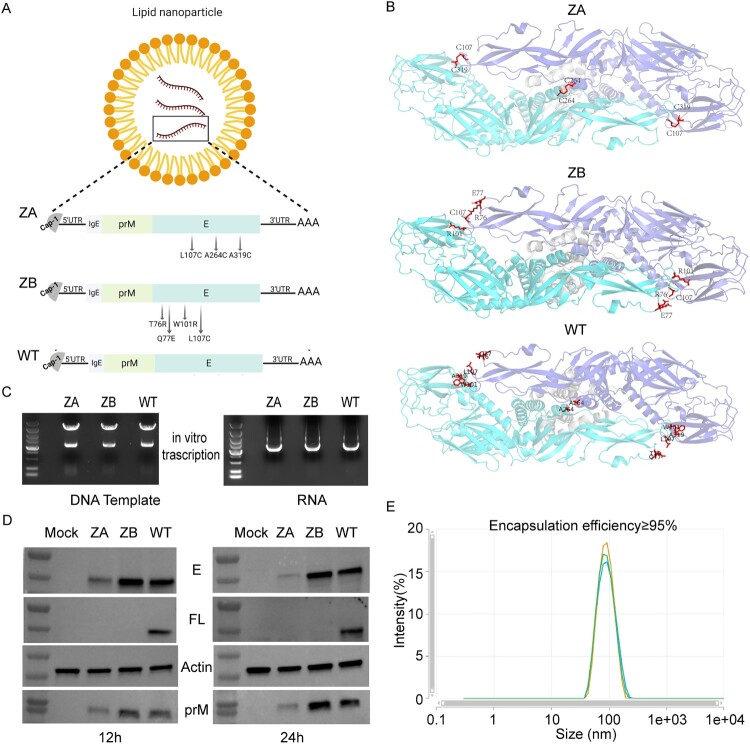
Design of ZIKV mRNA vaccines and in vitro expression. (A) Schematic illustration of ZIKV antigen sequences design and mRNA-LNPs (created with BioRender.com). (B) Results of structure prediction of prM and E proteins from ZA, ZB, and WT sequences (created with AlphaFold 3). (C) Agarose gel electrophoresis maps of linearized plasmids and transcribed mRNAs (5000 bp DNA marker). (D) Western-blot results of cellular protein expression of ZA, ZB, and WT mRNAs in Vero cells. (E) Particle size distribution of freshly prepared mRNA-LNPs

We aligned the amino acid sequences of over 300 ZIKV strains from both Asian and African lineages, selected the most conserved amino acid sequences of the prM and E proteins as target antigens, and designed two antigen sequences, ZA and ZB (Figure 1A). In ZA, there were three substitutions (L107C, A264C, A319C). In ZB, there were four substitutions (T76R, Q77E, W101R, L107C). WT, as a control, had no amino acid substitution. In silico prediction of immunogen structure, ZA, ZB and WT were predicted using AlphaFold 3 (https://alphafoldserver.com/) through the following protocol: First, the amino acid sequences of immunogens were input into the AlphaFold 3 web interface, followed by structural modelling using default parameters. The highest-scoring prediction was selected for structural visualization and analysis using PyMOL (v4.6.0), enabling detailed examination of molecular interactions and conformational characteristics [26]. The structure prediction revealed that the mutations in ZA facilitated the formation of three disulphide bonds, stabilized E protein dimers and shielded the FLEs, whereas the corresponding mutations in ZB directly destroyed the conserved FLEs (Figure 1B). Following plasmid linearization, we synthesized mRNAs via in vitro transcription (Figure 1C). Then, we transfected Vero cells with these mRNAs and demonstrated the intracellular expression of prM and E protein by western blot analysis. As shown in the figure (Figure 1D), we detected the prM and E proteins with the approximate molecular weight in cell lysates at 12 and 24 h post-transfection. Meanwhile, we did not recognize the fusion loop in ZA or ZB with mAb 4G2, which indicated that the function of the fusion loop was interfered. Subsequently, we prepared mRNA-LNPs using microfluidics. The freshly prepared LNPs exhibited an average size of approximately 100 nm, a polydispersity index of 0.1203, and an encapsulation efficiency exceeding 95% (Figure 1E), meeting the standards for in vivo RNA delivery.

### mRNA vaccines induced humoral and cellular immune responses in female C57BL/6 mice

**Figure 2.**
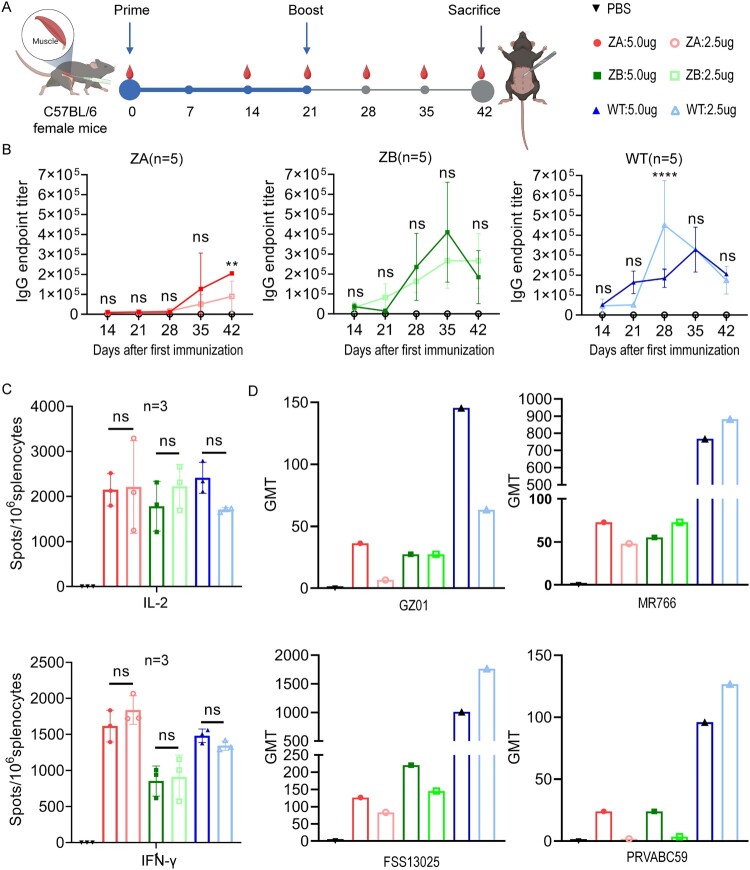
The immunogenicity of ZIKV mRNA vaccines in female C57BL/6 mice. (A) The timeline of vaccine immunization and sampling in female C57BL/6 mice (created with BioRender.com). (B) E protein-specific binding antibody levels at each time point after immunization in each group. Data are presented as mean ± SD (error bars). Two-way ANOVA: NS, not significant, *P* > 0.05; *P < 0.05; **P < 0.01; ***P < 0.001; and ****P < 0.0001. (C) The result of T cells secreting IL-2 and IFN-γ in each group at 21 days after booster immunization (n = 3). Data are presented as mean ± SD (error bars). Two-way ANOVA: NS, not significant. (D) The GMT of neutralization antibodies in each group at 21 days after booster immunization against GZ01, MR766, FSS13025, and PRVABC59 (n = 5).

To assess the immunogenicity of mRNA-LNPs, we immunized female C57BL/6 mice using a prime-boost strategy and collected serum samples throughout the immunization process (Figure 2A). Mice received either ZA, ZB or WT vaccines via intramuscular injection at low (2.5 μg) or high (5.0 μg) dose, while PBS-treated mice served as controls. After each immunization, there is no significant weight loss or adverse effects in any of the mice (Supplementary Figure S1). Meanwhile, we detected the levels of multiple cytokines in serum samples at 5 and 24 h post-immunization to evaluate instant immune response (Supplementary Figure S5). Compared to the PBS group, IL-6 and KC levels increased at 5 h post-immunization, while IL-5 levels decreased. To further evaluate humoral immunity, we measured antigen-specific immunoglobulin G (IgG) titres in mice serum by endpoint ELISA. All vaccine groups exhibited progressively increasing IgG levels compared to the PBS group after the first vaccination, with a significant boost following the second dose (Figure 2B). Compared to WT vaccine, ZA (5.0 μg) and ZB vaccines appeared different IgG changes, but with equivalent IgG level reaching to 2×10^5^ at 21 days post-booster immunization. Meanwhile, we detected the ZIKV-specific neutralizing antibodies (nAbs) titres in the circulation three weeks after the boost (Figure 2D). Compared to WT vaccine, ZA and ZB vaccines induced low levels of nAbs against Asian-lineage (FSS13025), African-lineage (MR766) and Asian/American-lineage (GZ01; PRVABC59) ZIKV strains. There was no significant difference in the nAbs levels induced by the same vaccine at different doses (Supplemental Figure S6A). To assess vaccine-induced cellular immunity, we characterized the antigen-specific responses of splenocytes in immunized C57BL/6 mice via ELISpot assay three weeks after the boost (Figure 2C and Supplementary Figure S2). With the stimulation of heat-inactivated ZIKV strain, four vaccine groups showed a significant increase in T lymphocytes secreting IL-2 and IFN-γ compared to the PBS group. The ZA group generated approximately 2000 IL-2-specific and 1700 IFN-γ-specific spots per 10^6^ splenocytes after stimulation, while the ZB group generated approximately 2000 IL-2-specific and 800 IFN-γ-specific spots per 10^6^ splenocytes. The results indicated that ZA could mobilize more immune T cells secreting IFN-γ.

### mRNA vaccines protected male and female AG129 mice from lethal ZIKV challenge at low levels of neutralizing antibodies

**Figure 3.**
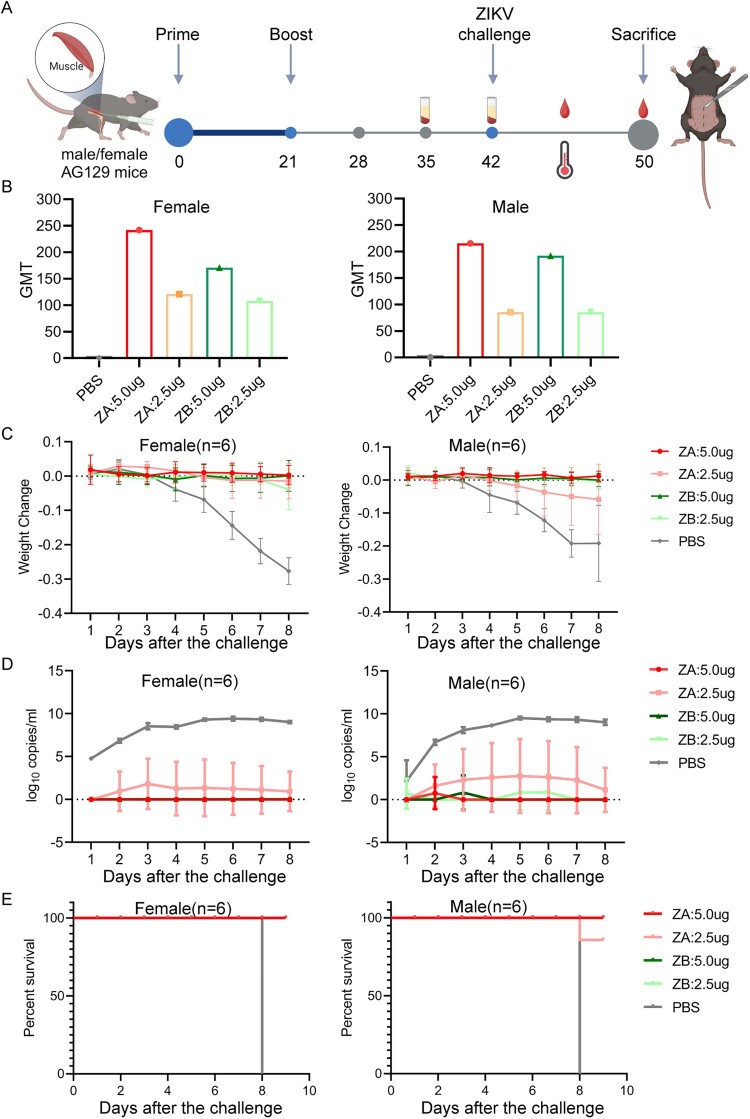
ZIKV mRNA vaccines protect male/female AG129 mice from lethal ZIKV challenge at low levels of neutralizing antibodies. (A) The timeline of vaccine immunization and ZIKV challenge in male/female AG129 mice (created with BioRender.com). (B) The GMT of neutralization antibodies of male/female AG129 mice in each group at 21 days after booster immunization against GZ01 (n = 6). (C) Weight change of male/female AG129 mice in each group after ZIKV challenge (n = 6). (D) Viral load in the blood of male/female AG129 mice in each group after the ZIKV challenge (n = 6). (E) Changes in survival rate of male/female AG129 mice in each group within 8 days of ZIKV infection (n = 6).

To evaluate the efficacy of the mRNA-LNPs against ZIKV, we used AG129 mice as the ZIKV challenge model. Mice were immunized with ZA or ZB vaccines using a prime-boost regimen and subsequently challenged with a lethal dose of ZIKV (10^3^ TCID_50_) via subcutaneous injection (Figure 3A). Neutralization assays performed one day before the challenge revealed low nAb titres against GZ01 in all vaccine groups, with GMTs not exceeding 250 for high-dose groups and 150 for low-dose groups (Figure 3B), which did not show the difference in the nAbs levels induced by the same vaccine at different doses except ZA vaccine in male mice (Supplemental Figure S6B). After the lethal challenge, we monitored the body weight of AG129 mice until all PBS mice died at 8 dpi (Figure 3C). ZA and ZB vaccines protected female AG129 mice from significant weight loss, while ZA vaccine (2.5 μg) only slowed down the weight loss in male mice. Meanwhile, we detected low-level viremia in male and female AG129 mice receiving the ZA vaccine (2.5 μg) (Figure 3D). However, viremia was absent in other vaccine groups by 8 days post-infection (dpi) (Figure 3D). Importantly, all mice immunized with 5.0 μg of ZA or ZB vaccine were fully protected against lethal ZIKV infection (Figure 3E). In contrast, PBS-treated mice exhibited severe symptoms, including ruffled fur, lethargy and a hunched posture at 3–4 dpi, leading to rapid deterioration and death at 8 dpi. Similar symptoms only appeared in individual male mice receiving the ZA vaccine (2.5 μg).

### ZA vaccine protected male and female AG129 mice from multi-tissue infection by ZIKV

**Figure 4.**
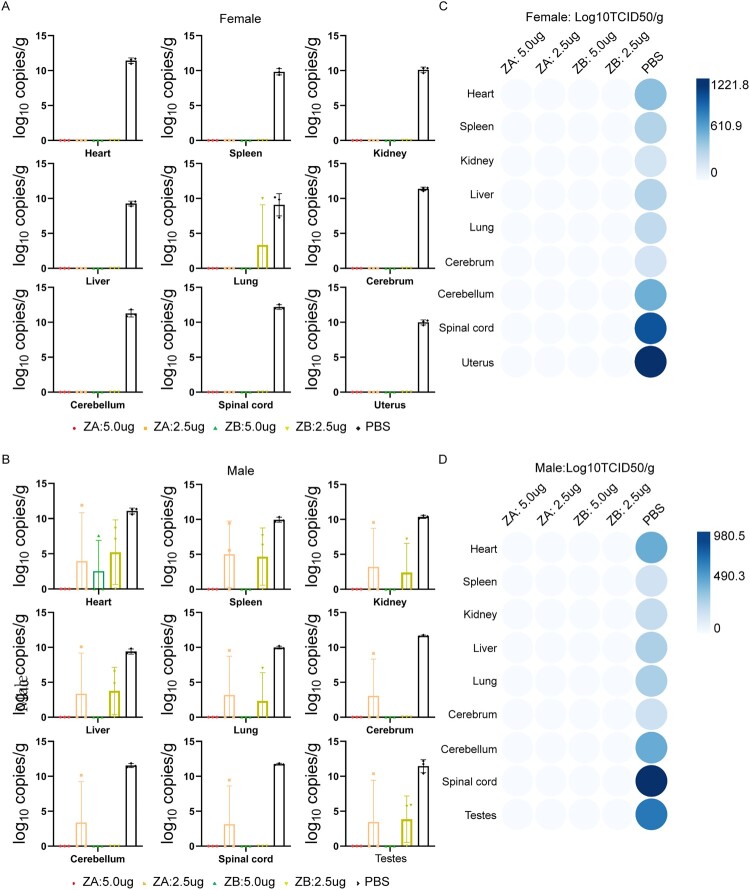
ZIKV mRNA vaccines protect male/female AG129 mice from multi-tissue infection by ZIKV. (A) Viral load in the heart, liver, spleen, lung, kidney, cerebrum, cerebellum, spinal cord, and uterus of female AG129 mice in each group (n = 3) at 8pi. (B) Viral load in the heart, liver, spleen, lung, kidney, cerebrum, cerebellum, spinal cord, and testicle of male AG129 mice in each group (n = 3) at 8 dpi. (C) Detection of infectious Zika virus in heart, liver, spleen, lung, kidney, cerebrum, cerebellum, spinal cord, and uterus of female AG129 mice in each group (n = 3) at 8 dpi. (D) Detection of infectious Zika virus in heart, liver, spleen, lung, kidney, cerebrum, cerebellum, spinal cord, and testicle of male AG129 mice in each group (n = 3) at 8 dpi.

ZIKV challenge resulted in viral infection and replication in multiple tissues of AG129 mice. To further assess the vaccine efficacy, we sacrificed all AG129 mice when the PBS-treated mice died of ZIKV infection at 8 dpi. We collected these critical tissue samples for the detection of viral load and infectious virus. Viral load from tissue samples was indicative of active virus replication (Figure 4A and B). The ZA vaccine (5.0 μg) could provide complete protection against multi-tissue infection in both male and female AG129 mice. However, the ZA vaccine (2.5 μg) and ZB vaccine (5.0 μg) could only provide partial protection in male AG129 mice, suggesting dose- or sex-dependent efficacy. No matter whether in female mice or male mice, the ZB vaccine (2.5 μg) did not completely prevent ZIKV infection in all these tissues. Notably, no infectious virus was detected in these tissues of the four vaccine groups compared to the PBS group (Figure 4C and D). As is known to all, ZIKV could cross the blood–brain, blood-testis and blood-placenta barriers. Compared to the PBS group, viral load and infectious virus were not detected in the cerebrum, cerebellum, testicle or uterus of mice immunized with ZA (5.0 μg) and ZB (5.0 μg) vaccines, highlighting their ability to block ZIKV dissemination across critical barriers.

### mRNA vaccine reduces pathological lesions caused by ZIKV challenge in male and female AG129 mice

**Figure 5.**
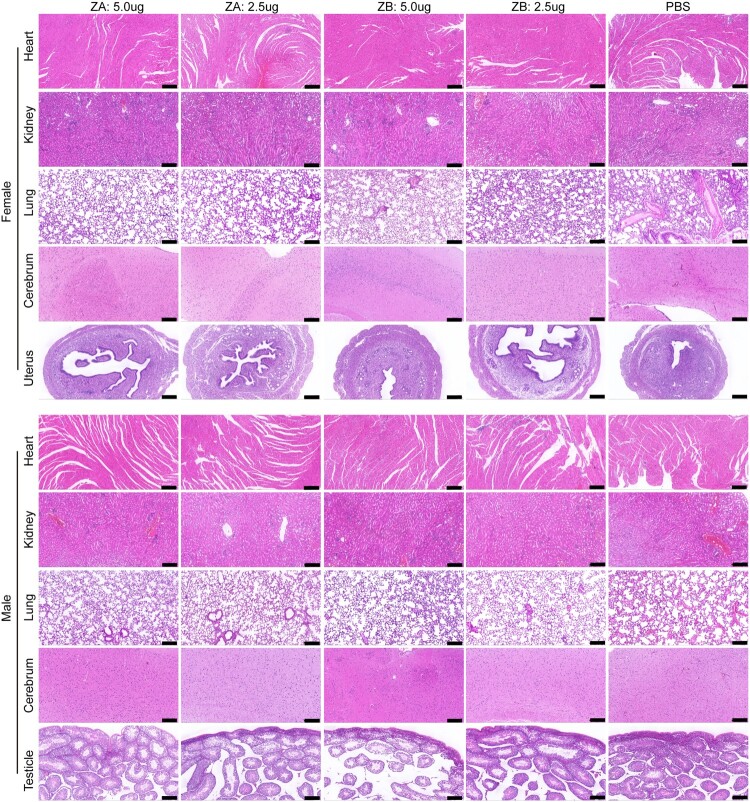
ZIKV mRNA vaccines could alleviate pathological lesions of the heart, kidney, lung, cerebrum, uterus or testicle of AG129 mice at 8 days post-ZIKV challenge (n = 3). The scale bar represents 200 μm.

To comprehensively evaluate the protective efficacy of vaccines, we collected critical tissue samples for pathological analysis. Compared to the PBS group, pathological lesions were significantly alleviated in all vaccine groups. According to the pathological outcome (Figure 5 and Supplementary Figure S4), symptoms of bleeding and inflammatory cell infiltration were milder in the heart, spleen, kidney and spinal cord of immunized mice compared to PBS-treated mice. Additionally, immunization significantly reduced the disappearance of germinal centres. In the liver, thrombosis and inflammatory cell infiltration were less severe in all vaccine groups than in the PBS group. Similarly, pneumorrhagia, inflammatory cell infiltration and bronchial blockage were milder in immunized mice. However, male AG129 mice in the ZA (5.0 μg) group exhibited more severe abnormal proliferation of alveolar type 2 cells and thickening of alveolar septa compared to the PBS group. In the cerebrum and cerebellum, local bleeding and microgliosis were less pronounced in all vaccine groups. For female AG129 mice, there is less inflammatory cell infiltration in uterus of all vaccine groups than the PBS group, with milder bleeding except in the ZB (5.0 μg) group. Male AG129 mice also exhibited milder bleeding in testes of all vaccine groups than the PBS group, with reduced inflammatory cell infiltration except in the ZB (5.0 μg) group. Pathological scores indicated that the ZA vaccine (5.0 μg) significantly alleviated lesions in the brain, uterus and testicle (Supplementary Figure S3).

### Immune serum showed reduced ADE activities

**Figure 6.**
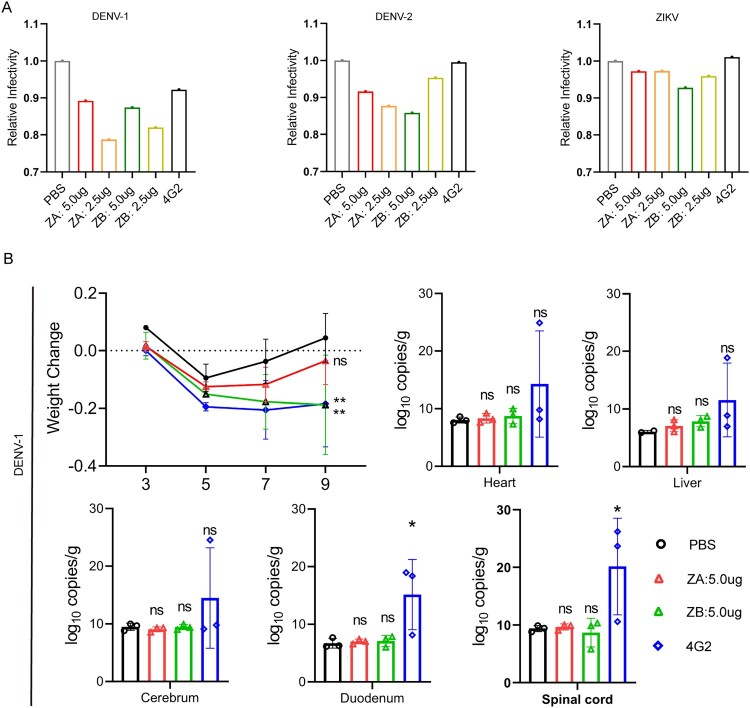
ZA and ZB vaccines reduced the enhancement of DENV infection. (A) Enhancing effects of ZIKV immune serum on DENV-1, DENV-2, and ZIKV infection on K562 cells. (B) Enhancing effects of ZIKV immune serum on DENV-1 infection in AG129 mice. Data are presented as mean±SD (error bars). Dunnett's multiple comparisons test with PBS group as the control: NS, not significant, *P* > 0.05; *P < 0.05.

To evaluate the in vitro ADE, we incubated diluted serum samples from female C57BL/6 mice with DENV or ZIKV to infect K562 cells. As a positive control, we used the pan-orthoflavivirus-specific monoclonal antibody 4G2, which binds the highly conserved flaviviral fusion loop epitopes [27]. Compared to the 4G2 group, the relative infectivity of DENV-1 and DENV-2 was lower in all vaccine groups (Figure 6A). Additionally, we also assessed the infection-enhancing activities of sera for ZIKV. Immunized serum did not show the ADE for ZIKV. These results demonstrated that the introduced mutations in the E protein reduced the production of antibodies enhancing dengue infection. Consistent with the ADE tendency in vitro, mice receiving 4G2 form succumbed to DENV-1 challenge significantly earlier than those receiving sera from the ZA-vaccinated donors. When mice receiving 4G2 lost nearly 20% weight, AG129 mice receiving sera from the ZA-based vaccines did not suffer from severe weight loss compared with those from the PBS group (Figure 6B). Meanwhile, no significant differences in viral load were observed in the duodenum or spinal cord of AG129 mice between the groups receiving sera from ZA/ZB-based vaccines and the PBS group.

#### Passive transfer of immune serum afforded partial protection against lethal ZIKV challenge

**Figure 7.**
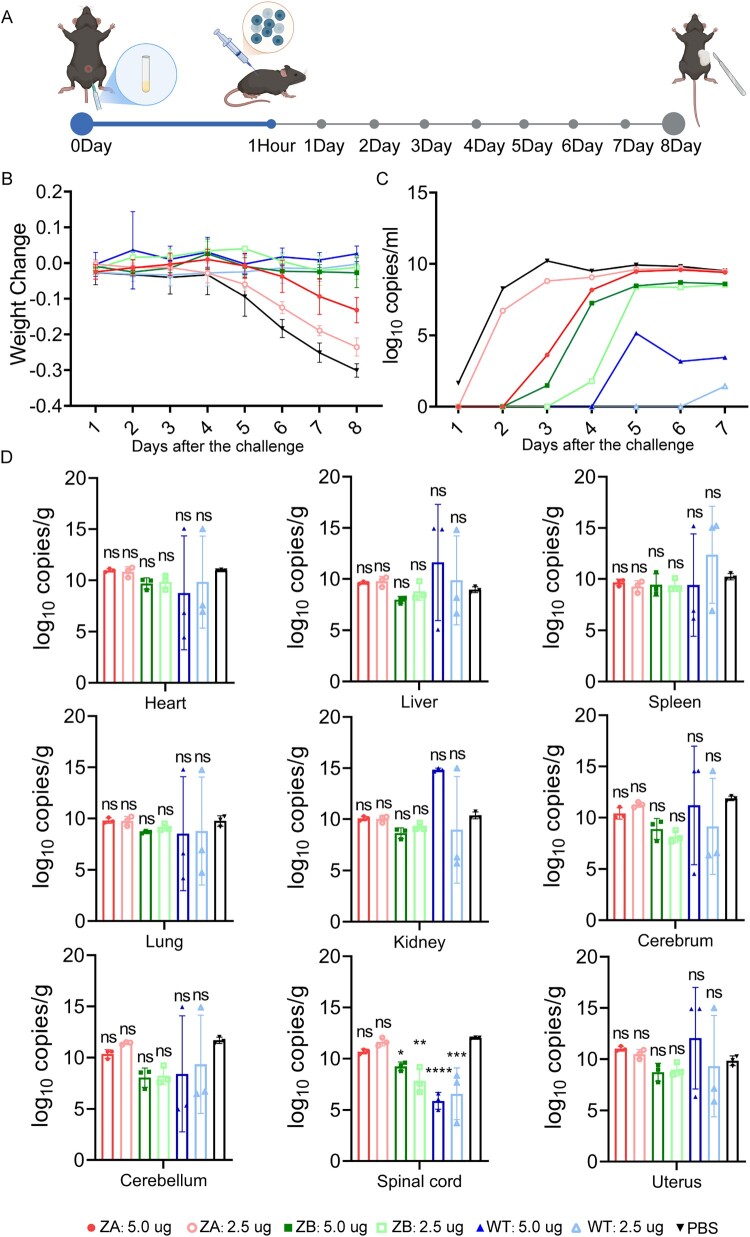
Passive transfer of immune serum afforded partial protection against lethal ZIKV challenge. (A) The timeline of passive transfer of immune serum in female AG129 mice (created with BioRender.com). (B) Weight change of female AG129 mice in each group after ZIKV challenge (n = 3). (C) Viral load in the blood of female AG129 mice in each group after ZIKV challenge (n = 3). (D) Viral load in the heart, liver, spleen, lung, kidney, cerebrum, cerebellum, spinal cord, and uterus of female AG129 mice in each group at 8 dpi (n = 3). Data are presented as mean±SD (error bars). Dunnett's multiple comparisons test with PBS group as the control: NS, not significant, *P* > 0.05; *P < 0.05; **P < 0.01; ***P < 0.001; and ****P < 0.0001.

To assess the efficacy of the immune serum, we performed passive transfer experiments in AG129 mice (Figure 7A). AG129 mice in PBS group died of ZIKV infection at 8 dpi. Compared to PBS group, mice that received the immune serum from ZB or WT vaccine did not appear significant weight loss at 8 dpi, while mice that received the immune serum from ZA (2.5 μg) vaccine lost more than 25% weight and that from ZA (5.0 μg) vaccine lost more than 10% weight (Figure 7B). Meanwhile, we detected the viral load in blood within 8 days after ZIKV challenge, which demonstrated that only AG129 mice in WT group appeared significantly reduced viremia compared to PBS group (Figure 7B). All AG129 mice receiving immune serum survived at 8 dpi, but viral load of most tissues showed no difference in immune serum group compared to PBS group (Figure 7C). These results indicated passive transfer of immune serum afforded partial protection against lethal ZIKV challenge.

## Discussion

In this study, we evaluated the protective efficacy of two mRNA vaccines encoding prM and E proteins in male and female AG129 mice. A prime-boost vaccination strategy protected AG129 mice from lethal ZIKV challenge and multi-tissue infection, indicating that our vaccine has the potential to respond rapidly to future ZIKV epidemics. Additionally, our ZIKV vaccines reduced the severity of dengue ADE induced by immunization.

The ZA vaccine (5.0 μg) demonstrated the best protection against ZIKV. Previously published anti-ZIKV vaccines have shown efficacy in mice or non-human primates (NHPs), protecting against viremia, tissue viral load, or lethal challenge [20,22,24,28,29]. Two ZIKV mRNA vaccines have entered Phase I trials, mRNA-1325 and mRNA-1893 from ModernaTX [23]. In mice experiment, mRNA-1325 showed poor immunogenicity. Although mRNA-1893 showed superior immunogenicity and efficacy compared with mRNA-1325 in rhesus macaques, both of them had no published experimental data about ADE evaluation. Additionally, ZIKV infection in mice or NHPs often leads to viral persistence in multiple tissues [30,31]. Unlike previous studies, which primarily detected viral infection in the blood or a few tissues, our study provided a more comprehensive analysis across nine tissues. The results demonstrated that the ZA vaccine (5.0 μg) achieved complete clearance of viral infection in AG129 mice.

It is noteworthy that ZA or ZB vaccines in AG129 mice produced higher levels of nAbs than those in C57 mice. This discrepancy may be attributed to the immune deficiency of AG129 mice. Compared to WT vaccine, ZA and ZB vaccine induced significantly lower levels of nAbs. The possible explanation is the substitution of amino acids in vaccine design, which caused insufficient exposure of neutralizing epitopes. When challenged with a lethal dose of ZIKV, AG129 mouse model exhibited sex-based differences in protection efficacy, particularly at the ZA vaccine (2.5 μg) and ZB vaccine (2.5 μg). Females exhibited the complete viral clearance, possibly due to estrogen-enhanced immunity or stronger Th2-type immune responses [32,33]. Additionally, the ZA vaccine (5.0 μg) only partially alleviated pathological lesions in certain tissues. According to our previous findings [31], ZIKV infection triggers a massive release of cytokines in AG129 mice, suggesting that pathological damage may be linked to excessive immune cell activation following ZIKV challenge.

To better understand the humoral responses and correlates of protection induced by the mRNA vaccines, we performed passive protection studies in AG129 mice. The results demonstrated that passive transfer of immune serum could not protect AG129 mice from lethal ZIKV infection. The main reason for this phenomenon might be that T-cell immunity of the vaccine played a major antiviral protective role [34,35]. In C57BL/6 mice, ZA vaccines induced higher levels of T cells secreting IFN-γ by ELISpot assay than ZB vaccines. In this study, passive immunization could only provide low levels of nAbs in immune serum, without the cooperation of T cell immunity. During active immunity, ZIKV-specific T cell responses could compensate for low levels of humoral immunity. Additionally, passive antibody injection, without IFN signal amplification, finds it difficult to suppress viral infection in AG129 mice.

The ZA and ZB vaccines showed reduced ADE in vitro. Previous studies have described that certain mutations, such as W101R, in ZIKV vaccines significantly reduced the ADE of dengue infection [24,25]. However, these mutations often compromise neutralizing antibody titres and provide incomplete protection against viremia and tissue infection [24,25]. The W101 mutation disturbed E-dimer formation and the E-dimer epitope (EDE), which is targeted by potent ZIKV nAbs [25,36]. Maintaining the E-dimer conformation is likely critical for vaccine efficacy. Therefore, we introduced three disulphide bonds in the ZA antigen. Although the ZA vaccine (5.0 ug) also induced low nAb levels in mice, it provided complete protection and prevented multi-tissue infection in AG129 mice without enhancing dengue infection. Although the NS1-based vaccine could avoid ADE, the viral clearance efficiency in the mouse model is significantly lower than that of prM-E vaccine [37,38]. However, the ZA mRNA vaccine has some limitations. First, the protection efficacy has only been tested in AG129 mice. Although AG129 mice have been used extensively for the evaluation of short-term protection efficacy of ZIKV vaccine, they could not fully simulate the pathological processes of immunocompetent hosts. Since non-human primates (NHPs) are the natural hosts of ZIKV, further evaluation in NHPs is necessary. Second, maternal ZIKV infection during pregnancy is associated with congenital malformations in the fetus [39], so it is essential to determine whether the vaccine could block vertical transmission of ZIKV. Finally, we only reported the vaccine's short-term protection. The durability of vaccine-induced immunity requires further investigation. Future studies will address these issues. In conclusion, the modified mRNA vaccine elicited sufficient immunity, protecting against ZIKV infection and disease in AG129 mice.

## Supplementary Material

Supplementary_material-clean.docx

## Data Availability

All the data supporting the findings of this current study are available from the article and/or supplemental material.

## References

[1] Vue D, Tang Q. Zika virus overview: transmission, origin, pathogenesis, animal model and diagnosis. Zoonoses. 2021;1(1). doi:10.15212/zoonoses-2021-0017 PMC8698461 34957474

[2] Dick GW, Kitchen SF, Haddow AJ. Zika virus. I. isolations and serological specificity. Trans R Soc Trop Med Hyg. 1952 Sep;46(5):509–520. doi:10.1016/0035-9203(52)90042-4 12995440

[3] Weaver SC, Costa F, Garcia-Blanco MA, et al. Zika virus: history, emergence, biology, and prospects for control. Antiviral Res. 2016 Jun;130:69–80. doi:10.1016/j.antiviral.2016.03.010 26996139 PMC4851879

[4] Pettersson JH, Eldholm V, Seligman SJ, et al. How did Zika virus emerge in the Pacific islands and Latin America? mBio. 2016 Oct 11;7(5):e01239–16. doi:10.1128/mBio.01239-16 27729507 PMC5061869

[5] Liu Y, Liu J, Du S, et al. Evolutionary enhancement of Zika virus infectivity in Aedes aegypti mosquitoes. Nature. 2017 May 25;545(7655):482–486. doi:10.1038/nature22365 28514450 PMC5885636

[6] Song GY, Huang XY, He MJ, et al. A single amino acid substitution in the capsid protein of Zika virus contributes to a neurovirulent phenotype. Nat Commun. 2023 Oct 26;14(1):6832. doi:10.1038/s41467-023-42676-7 37884553 PMC10603150

[7] Yuan L, Huang XY, Liu ZY, et al. A single mutation in the prM protein of Zika virus contributes to fetal microcephaly. Science. 2017 Nov 17;358(6365):933–936. doi:10.1126/science.aam7120 28971967

[8] Cao-Lormeau VM, Blake A, Mons S, et al. Guillain-Barré syndrome outbreak associated with Zika virus infection in French Polynesia: a case-control study. Lancet. 2016 Apr 9;387(10027):1531–1539. doi:10.1016/S0140-6736(16)00562-6 26948433 PMC5444521

[9] Paploski IA, Prates AP, Cardoso CW, et al. Time lags between exanthematous illness attributed to Zika virus, Guillain-Barré syndrome, and microcephaly, Salvador, Brazil. Emerg Infect Dis. 2016 Aug;22(8):1438–1444. doi:10.3201/eid2208.160496 27144515 PMC4982160

[10] Ikejezie J, Shapiro CN, Kim J, et al. Zika virus transmission - region of the americas, May 15, 2015-December 15, 2016. MMWR Morb Mortal Wkly Rep. 2017 Mar 31;66(12):329–334. doi:10.15585/mmwr.mm6612a4 28358795 PMC5657956

[11] Sun H, Dickens BL, Jit M, et al. Mapping the cryptic spread of the 2015-2016 global Zika virus epidemic. BMC Med. 2020 Dec 17;18(1):399. doi:10.1186/s12916-020-01845-x 33327961 PMC7744256

[12] Mukhopadhyay S, Kuhn RJ, Rossmann MG. A structural perspective of the flavivirus life cycle. Nat Rev Microbiol. 2005 Jan;3(1):13–22. doi:10.1038/nrmicro1067 15608696

[13] Kuno G, Chang GJ. Full-length sequencing and genomic characterization of Bagaza, Kedougou, and Zika viruses. Arch Virol. 2007;152(4):687–696. doi:10.1007/s00705-006-0903-z 17195954

[14] Agrelli A, de Moura RR, Crovella S, et al. Zika virus entry mechanisms in human cells. Infect Genet Evol. 2019 Apr;69:22–29. doi:10.1016/j.meegid.2019.01.018 30658214

[15] Shi Y, Gao GF. Structural biology of the Zika virus. Trends Biochem Sci. 2017 Jun;42(6):443–456. doi:10.1016/j.tibs.2017.02.009 28318966

[16] Zhang Y, Corver J, Chipman PR, et al. Structures of immature flavivirus particles. EMBO J. 2003 Jun 2;22(11):2604–2613. doi:10.1093/emboj/cdg270 12773377 PMC156766

[17] Prasad VM, Miller AS, Klose T, et al. Structure of the immature Zika virus at 9 Å resolution. Nat Struct Mol Biol. 2017 Feb;24(2):184–186. doi:10.1038/nsmb.3352 28067914 PMC5296287

[18] Dai L, Song J, Lu X, et al. Structures of the Zika virus envelope protein and Its complex with a flavivirus broadly protective antibody. Cell Host Microbe. 2016 May 11;19(5):696–704. doi:10.1016/j.chom.2016.04.013 27158114

[19] Dowd KA, DeMaso CR, Pelc RS, et al. Broadly neutralizing activity of Zika virus-immune sera identifies a single viral serotype. Cell Rep. 2016 Aug 9;16(6):1485–1491. doi:10.1016/j.celrep.2016.07.049 27481466 PMC5004740

[20] Dowd KA, Ko SY, Morabito KM, et al. Rapid development of a DNA vaccine for Zika virus. Science. 2016 Oct 14;354(6309):237–240. doi:10.1126/science.aai9137 27708058 PMC5304212

[21] Dai L, Xu K, Li J, et al. Protective Zika vaccines engineered to eliminate enhancement of dengue infection via immunodominance switch. Nat Immunol. 2021 Aug;22(8):958–968. doi:10.1038/s41590-021-00966-6 34267374

[22] Pardi N, Hogan MJ, Pelc RS, et al. Zika virus protection by a single low-dose nucleoside-modified mRNA vaccination. Nature. 2017 Mar 9;543(7644):248–251. doi:10.1038/nature21428 28151488 PMC5344708

[23] Bollman B, Nunna N, Bahl K, et al. An optimized messenger RNA vaccine candidate protects non-human primates from Zika virus infection. NPJ Vaccines. 2023 Apr 20;8(1):58. doi:10.1038/s41541-023-00656-4 37080988 PMC10119314

[24] Richner JM, Himansu S, Dowd KA, et al. Modified mRNA vaccines protect against Zika virus infection. Cell. 2017 Mar 9;168(6):1114–1125.e10. doi:10.1016/j.cell.2017.02.017 28222903 PMC5388441

[25] Slon-Campos JL, Dejnirattisai W, Jagger BW, et al. A protective Zika virus E-dimer-based subunit vaccine engineered to abrogate antibody-dependent enhancement of dengue infection. Nat Immunol. 2019 Oct;20(10):1291–1298. doi:10.1038/s41590-019-0477-z 31477918 PMC6839414

[26] Abramson J, Adler J, Dunger J, et al. Accurate structure prediction of biomolecular interactions with AlphaFold 3. Nature. 2024 Jun;630(8016):493–500. doi:10.1038/s41586-024-07487-w 38718835 PMC11168924

[27] Shukla R, Beesetti H, Brown JA, et al. Dengue and Zika virus infections are enhanced by live attenuated dengue vaccine but not by recombinant DSV4 vaccine candidate in mouse models. EBioMedicine. 2020 Oct;60:102991. doi:10.1016/j.ebiom.2020.102991 32949997 PMC7501058

[28] Abbink P, Larocca RA, De La Barrera RA, et al. Protective efficacy of multiple vaccine platforms against Zika virus challenge in rhesus monkeys. Science. 2016 Sep 9;353(6304):1129–1132. doi:10.1126/science.aah6157 27492477 PMC5237380

[29] Larocca RA, Abbink P, Peron JP, et al. Vaccine protection against Zika virus from Brazil. Nature. 2016 Aug 25;536(7617):474–478. doi:10.1038/nature18952 27355570 PMC5003703

[30] Hirsch AJ, Smith JL, Haese NN, et al. Zika virus infection of rhesus macaques leads to viral persistence in multiple tissues. PLoS Pathog. 2017 Mar 9;13(3):e1006219. doi:10.1371/journal.ppat.1006219 28278237 PMC5344528

[31] Yan Y, Yang H, Yang Y, et al. The inoculum dose of Zika virus can affect the viral replication dynamics, cytokine responses and survival rate in immunocompromised AG129 mice. Mol Biomed. 2024 Aug 3;5(1):30. doi:10.1186/s43556-024-00195-x 39095588 PMC11297010

[32] Klein SL, Marriott I, Fish EN. Sex-based differences in immune function and responses to vaccination. Trans R Soc Trop Med Hyg. 2015 Jan;109(1):9–15. doi:10.1093/trstmh/tru167 25573105 PMC4447843

[33] Girón-González JA, Moral FJ, Elvira J, et al. Consistent production of a higher TH1:TH2 cytokine ratio by stimulated T cells in men compared with women. Eur J Endocrinol. 2000 Jul;143(1):31–36. doi:10.1530/eje.0.1430031 10870028

[34] Tai W, Feng S, Chai B, et al. An mRNA-based T-cell-inducing antigen strengthens COVID-19 vaccine against SARS-CoV-2 variants. Nat Commun. 2023 May 23;14(1):2962. doi:10.1038/s41467-023-38751-8 37221158 PMC10204679

[35] Karl V, Hofmann M, Thimme R. Role of antiviral CD8+ T cell immunity to SARS-CoV-2 infection and vaccination. J Virol. 2025 Apr 15;99(4):e0135024. doi:10.1128/jvi.01350-24 40029063 PMC11998524

[36] Barba-Spaeth G, Dejnirattisai W, Rouvinski A, et al. Structural basis of potent Zika-dengue virus antibody cross-neutralization. Nature. 2016 Aug 4;536(7614):48–53. doi:10.1038/nature18938 27338953

[37] Zhan Y, Pang Z, Du Y, et al. NS1-based DNA vaccination confers mouse protective immunity against ZIKV challenge. Infect Genet Evol. 2020 Nov;85:104521. doi:10.1016/j.meegid.2020.104521 32882433

[38] Brault AC, Domi A, McDonald EM, et al. A Zika vaccine targeting NS1 protein protects immunocompetent adult mice in a lethal challenge model. Sci Rep. 2017 Nov 7;7(1):14769. doi:10.1038/s41598-017-15039-8 29116169 PMC5677088

[39] Brasil P, Pereira JP Jr, Moreira ME, et al. Zika virus infection in pregnant women in Rio de janeiro. N Engl J Med. 2016 Dec 15;375(24):2321–2334. doi:10.1056/NEJMoa1602412 26943629 PMC5323261

