# Supplementary material for: The modified mRNA vaccine protects immunocompromised AG129 mice from lethal challenge and multi-tissue infection by Zika virus
Source: Emerg Microbes Infect. 2025 Sep 3;14(1):2556729. doi: 10.1080/22221751.2025.2556729 (PMC12451962; doi:10.1080/22221751.2025.2556729)
Supplement: Supplementary_material-clean.docx [file TEMI_A_2556729_SM4347.docx]

**The modified mRNA vaccine protects immunocompromised AG129 mice from lethal challenge and multi-tissue infection by Zika virus**

**Supplementary Figures**

**
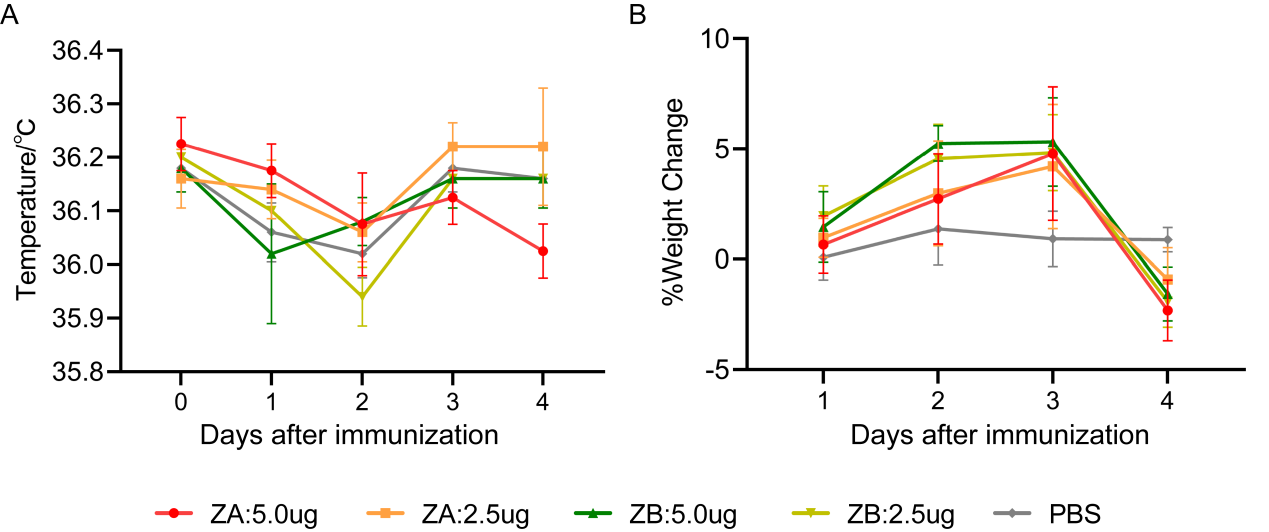
**

**Supplementary Figure S1**. Line chart of weight change (A) and body temperature (B) of female C57BL/6 mice in each group after prime immunization (n=5).

**
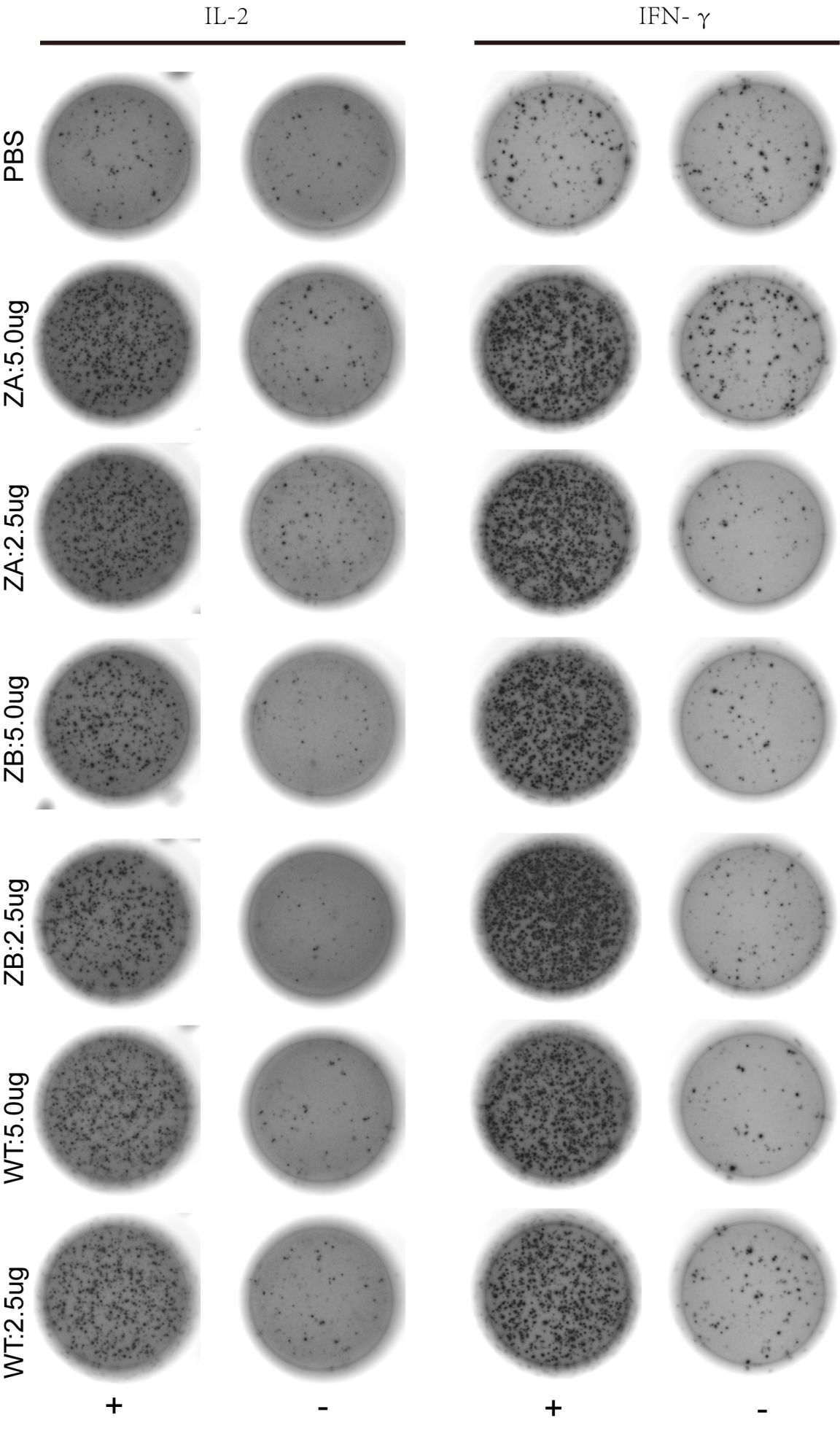
**

**Supplementary Figure S2.** T cells secreting IL-2 and IFN-γ in C57BL/6 mice group at 21 days after booster immunization.

**
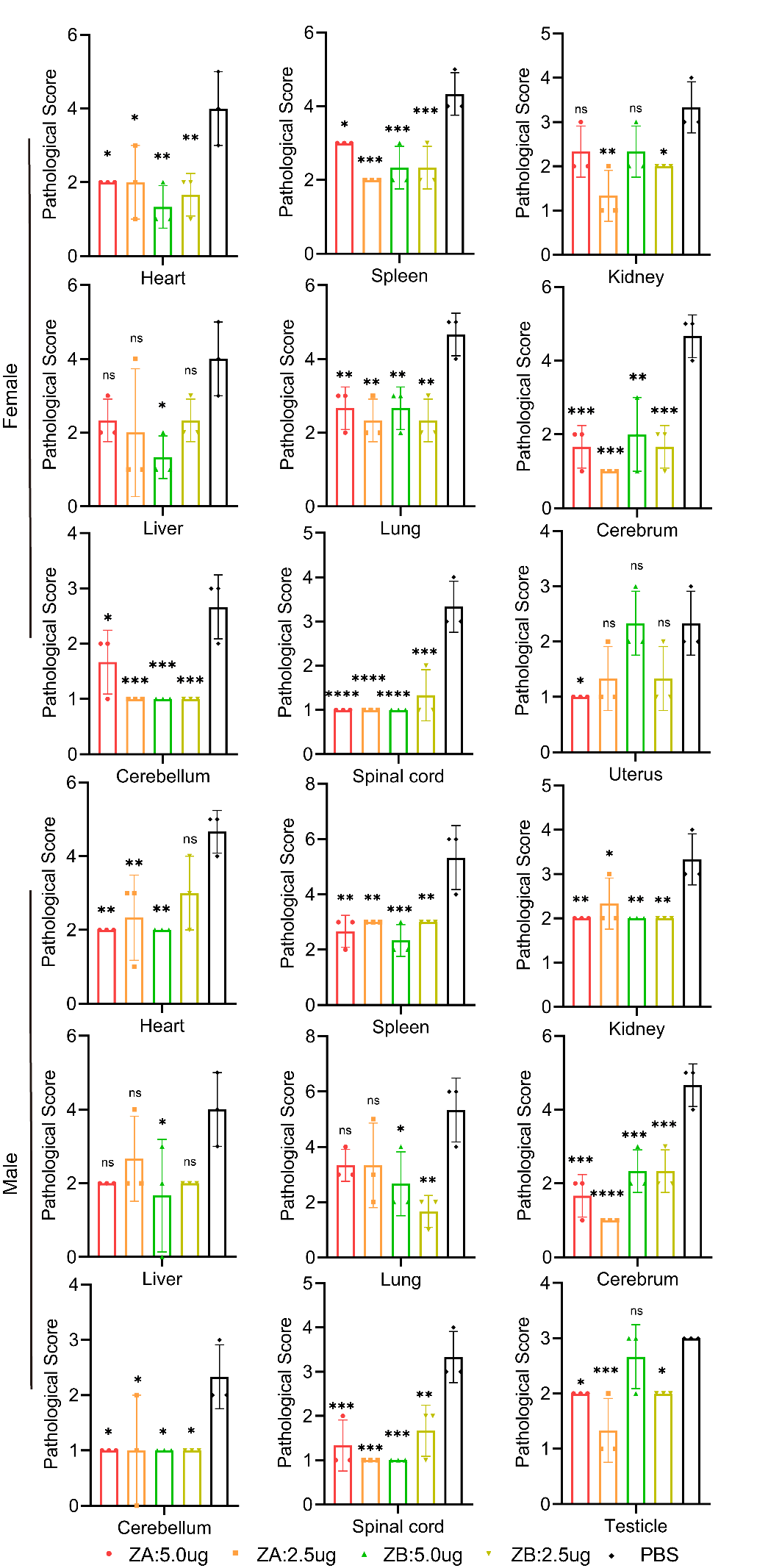
**

**Supplementary Figure S3.** The pathological score of heart, liver, spleen, lung, kidney, cerebrum, cerebellum, spinal cord, testicle or uterus of AG129 mice at 8 days post-ZIKV challenge (n=3). Dunnett's multiple comparisons test with PBS group as the control: NS, not significant, P > 0.05; *P < 0.05; **P < 0.01; ***P < 0.001; and ****P < 0.0001.


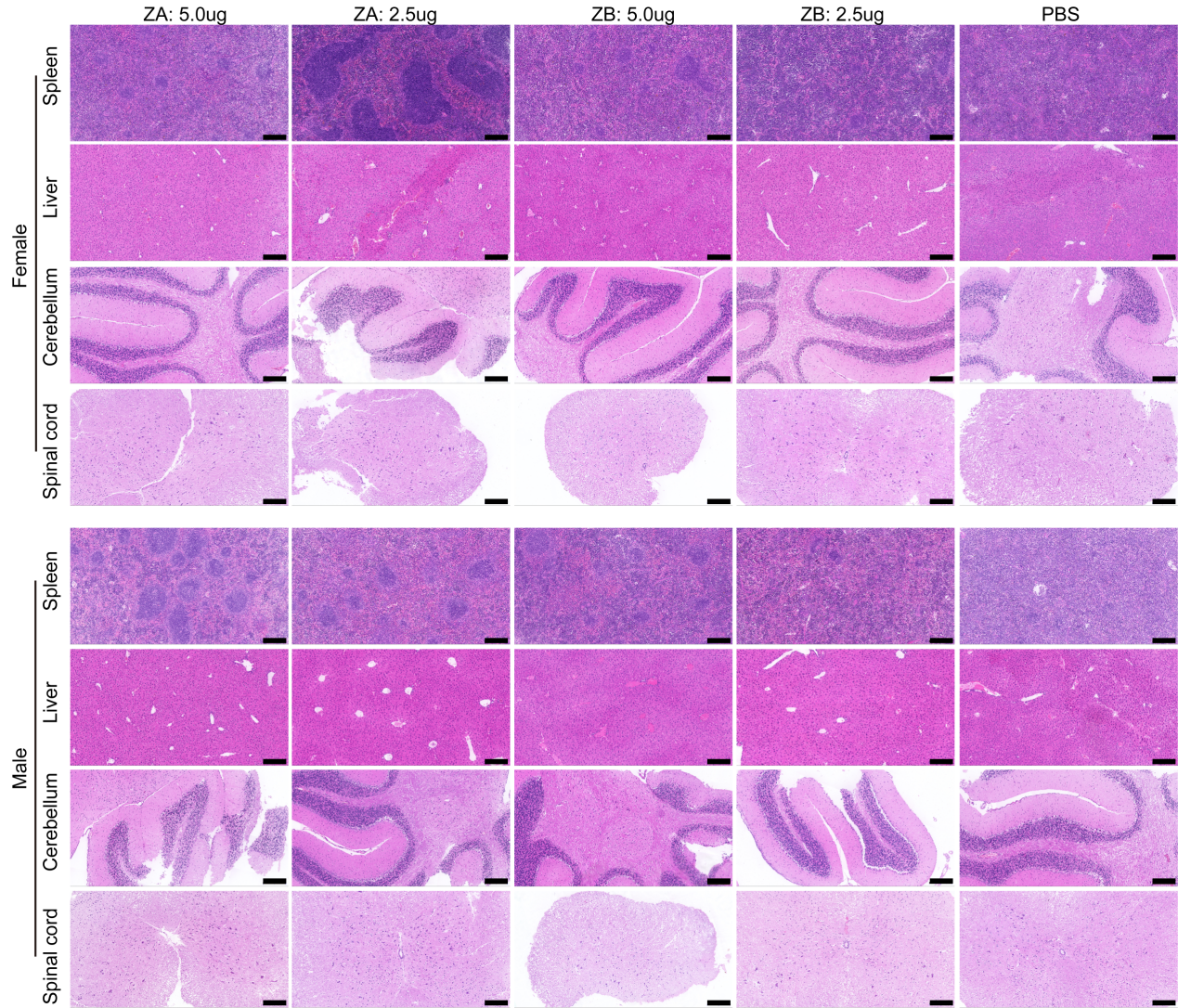


**Supplement Figure S4**. ZIKV mRNA vaccines could alleviate pathological lesions of the spleen, liver, cerebellum and spinal cord of AG129 mice at 8 days post-ZIKV challenge (n=3). The scale bar represents 200 μm.


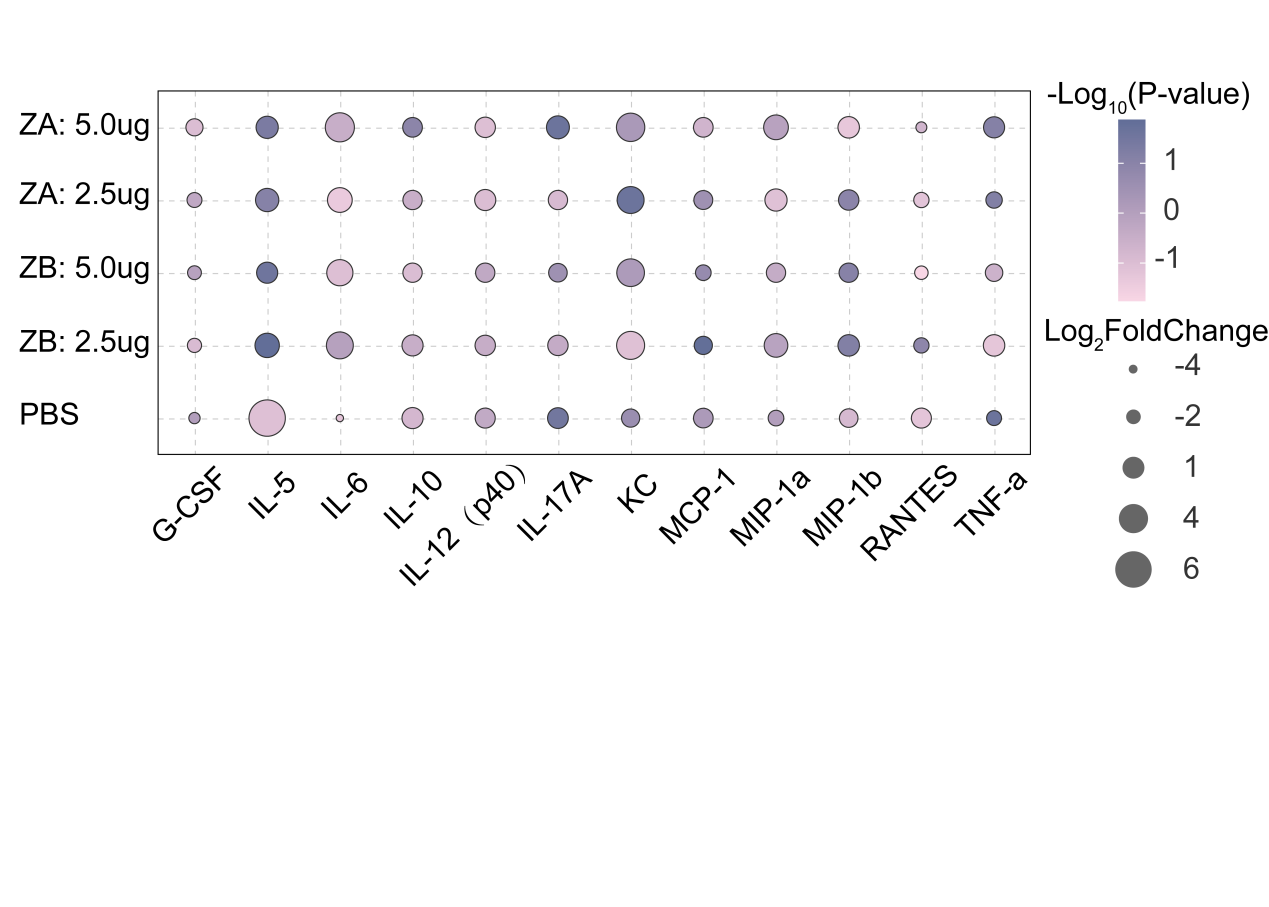


**Supplement Figure S5**. Fold change in the levels of inflammation-related cytokines in the serum at 5 hours and 24 hours after immunization (n=4). The color of the circle represents the P-value. The smaller the P value is, the darker the color. When P<0.05, -Log_10_(P)>1.3. The size of the circle represents the fold change (FC). FC = cytokine level at 5 hours after immunization /cytokine level at 24 hours after immunization. The greater the FC is, the larger the circle.


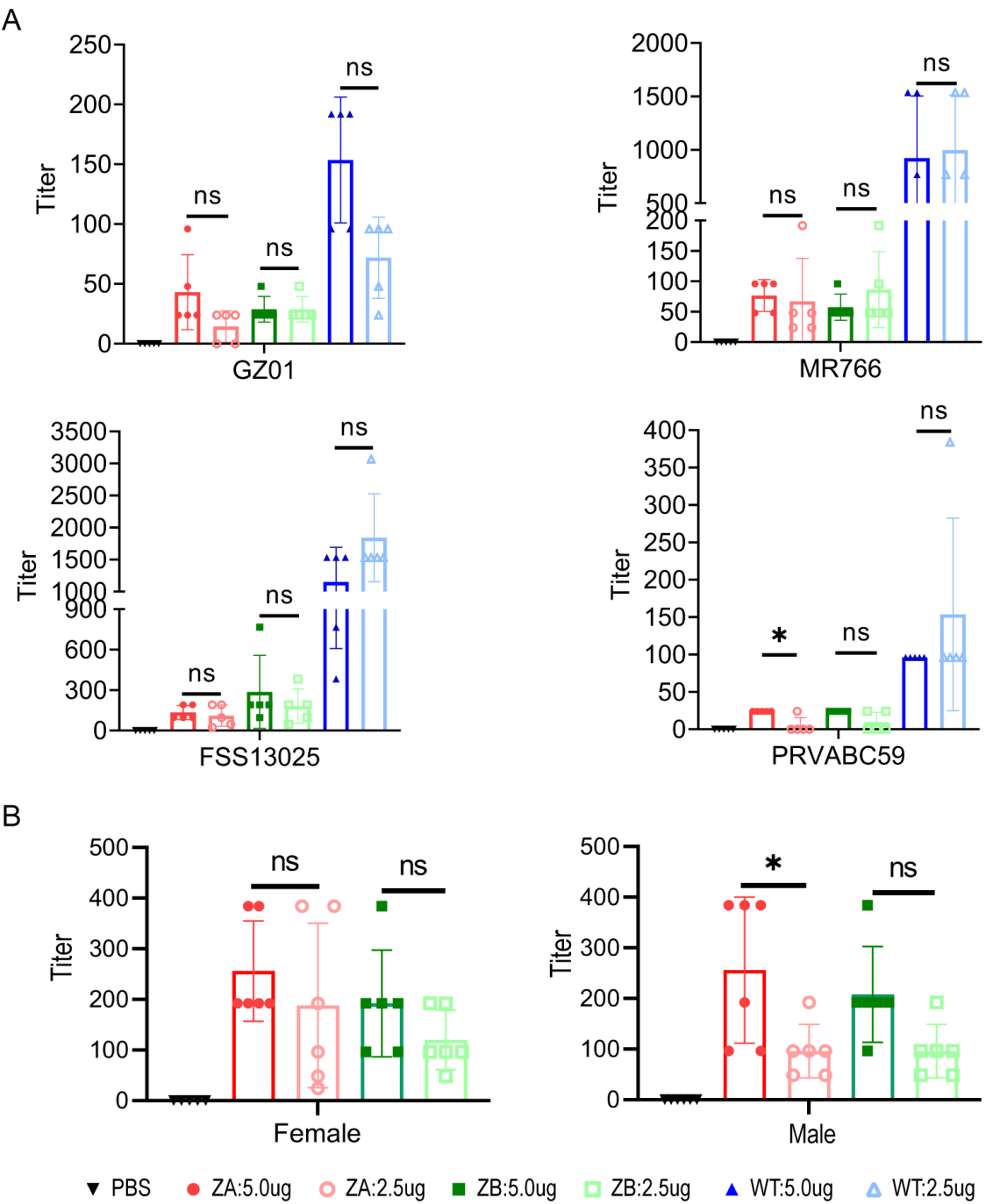


**Supplement Figure S6**. (A) Neutralization antibodies titer of female C57BL/6 mice in each group at 21 days after booster immunization against GZ01, MR766, FSS13025 and PRVABC59 (n=5); (B) Neutralization antibodies titer of male/female AG129 mice in each group at 21 days after booster immunization against GZ01 (n=6); Tukey's multiple comparisons test: NS, not significant, P > 0.05; *P < 0.05.

**Materials and methods**

***Cell and virus***

Vero cells were maintained in our laboratory and cultured in Dulbecco’s modified Eagle medium (DMEM; Gibco) supplemented with 10% fetal bovine serum (FBS; Gibco) and 1% penicillin/streptomycin at 37℃ in a humidified incubator with 5% CO_2_. K562 cells were purchased from Procell Life Science & Technology and cultured under the same conditions. The ZIKV strain GZ01 (GenBank: KU820898) was obtained from Institute of Medical Biology, Chinese Academy of Medical Science. The ZIKV strain MR766, FSS13025 and PRVABC59 were obtained from Institute of Microbiology, Chinese Academy of Sciences. DENV-1 (GenBank: PV162554) was obtained from Institute of Medical Biology, Chinese Academy of Medical Science. DENV-2 was obtained from the Third Affiliated Hospital of Guangzhou Medical University.

***Neutralization assay***

To evaluate the neutralizing antibody levels in mouse serum, we performed twelve 2-fold serial dilutions of serum at an initial ratio of 1:16 (serum: DMEM) in 96-well plates. Then, 200 TCID_50_ of ZIKV was added to every well. After incubating these plates for 1 hour at 37℃, we added 1.2×10^4^ Vero cells to each well. Finally, plates were incubated for six days at 37°C with 5% CO_2_. We evaluated the neutralizing ability of the serum sample by cytopathogenic effects (CPE). The Karber method was used to calculate the neutralizing antibodies titers, which were the highest serum dilution resulting in 50% neutralization inhibition.

***Enzyme-linked immunosorbent assay***

We collected serum samples at regular intervals during immunization. ZIKV E protein (AntibodySystem, YVV31401) was immobilized on a plate, and serum was serially diluted and added to the plate. A conjugated secondary antibody (Thermo, A10668) was then added at a dilution of 1:10000. The 1-Step™ Ultra TMB-ELISA substrate (Thermo, 34028) produced a signal proportional to the amount of antibody bound to the antigen in the well. Finally, plates were read using an enzyme-labelled instrument (BioTek), and data were processed based on the OD values.

***Enzyme-linked immunospot (ELISpot) assay***

To evaluate cell-mediated immunity, we used Mouse IL-2 and IFN-γ kit (MABTECH, 3441-4APW-10 and 3321-4APW-10). Peripheral blood mononuclear cells (PBMCs) were isolated from the spleens of immunized mice and added to ELISpot plates. ZIKV strain GZ01 was used as an antigenic stimulus. The titer of GZ01 was 10^6^ TCID_50_/ml. During the assay, the virus was inactivated at 56℃ for 30 min and aspirated 2 μ l into each well. Then, the plates were incubated for 36 hours at 37℃ with 5% CO_2_. To detect spots, we added detection antibody, Streptavidin-ALP, and substrate solution according to the kit instructions. Finally, spots were counted using an ELISpot reader.

***Viral titration***

Viral titers were determined using the median tissue culture infective dose (TCID_50_) in Vero cells. We performed six 10-fold serial dilutions of tissue homogenates in DMEM. After the cells were inoculated into 96-well plates, the plates were incubated for six days at 37°C with 5% CO_2_. The TCID_50_ was calculated using the Spearman-Karber method.

***Histopathology***

Tissue samples were fixed in 10% formalin, embedded in paraffin, and sectioned at 5-μm. Sections were stained with hematoxylin and eosin (HE) and scanned using 3DHISTECH. H&E-stained slides were scored by an experienced pathologist using CaseViewer software.

***Cytokine measurement***

Levels of immune-related cytokines were measured using a Bio-Plex Pro Mouse Cytokine 23-plex Assay (#M60009RDPD) following the manufacturer's instructions. Serum samples were allowed to clot for at least one hour at room temperature before centrifugation at 3500 rpm for 15 minutes. Coupled beads, samples, standards, blanks, detection antibodies, and streptavidin-phycoerythrin were added to each well, and plates were read immediately.
